# The Effect of Additional Pleural Procedures onto Recurrence Rates on the Spontaneous Pneumothorax Surgery

**DOI:** 10.5812/ircmj.7990

**Published:** 2013-02-05

**Authors:** Arife Zeybek, Serdar Kalemci, Özlem Gürünlü Alma, Alev Süzen, Murat Akgül, Kadir Koç

**Affiliations:** 1 Mugla Sıtkı Koçman University Medical Faculty, Chest Surgery Clinic, Mugla, Turkey; 2 Mugla Sıtkı Koçman University Medical Faculty, Chest Diseases Clinic, Mugla, Turkey; 3 Mugla Sıtkı Koçman University, Faculty of Sciences, Department of Statistics, Mugla, Turkey; 4 Mugla State Hospital, Pediatric Surgery Clinic, Mugla, Turkey

**Keywords:** Pneumothorax, Primary Spontaneous, Thoracotomy

## Abstract

**Background:**

Spontaneous pneumothoraxes constituted 1/1000 hospital admissions. They are particularly one of life threatening health issues in combination with bilateral pneumothorax, tension pneumothorax, repertory failure or COPD.

**Objectives:**

The cases of spontaneous pneumothorax represent a significant portion of the patients profile within the chest surgery clinics. The risk of recurrent pneumothorax in post thoracoscopy is between 2% and 14%, thus the subject of cure treatment and approach is still controversial. The cases were retrospectively treated due to spontaneous pneumothorax and their reasons, treatment approaches and results were comparatively examined with the literature.

**Patients and Methods:**

The years between 2007 and 2010, according to our hospital clinic, outpatients and accident &amp; emergency admission records, 79 patients were admitted with a diagnosis of spontaneous pneumothorax; and the patients’ age, gender, symptoms, types of pneumothorax, surgical intervention and recurrence, average length of stay, mortality and complications were retrospectively evaluated.

**Results:**

Seventy of all the patients (88.6%) were male and 9 of those (13.7%) were female. The mean age was calculated as 45.50 ± 21.07 (0-85). The patients were comprised of 41 (51.9%) with primary spontaneous pneumothorax and 38 (48.1%) with secondary spontaneous pneumothorax. 55 of the patients (69.6%) with the first attack, and 24 patients (30.4%) with post tube thoracotomy’s 2nd or 3rd pneumothorax attack were admitted. Those who were accepted with post tube thoracostomy’s 2nd or 3rd attack made up 2/3 of the secondary spontaneous pneumothorax patients. 57 of the patients (68.4%) were treated with the tube thoracostomy. The tube thoracostomy related complication was 6.3%, hemorrhage due to parenchymal damage and massive air leak were observed. An open surgical method to 22 of those patients and apical resection and apical pleurectomy + tetracycline pleurodesis to 16 of whom and bullae ligation and mechanical abrasion to 6 patients were applied. The recurrence of pneumothorax in post-surgery was not observed for 1-3 year period Complication was not detected .Mortality, one patient (1.3%) died in post tube thoracotomy, which was a stage 4 lung cancer patient.

**Conclusions:**

Most cases for pneumothorax were consisted of the patients with the primary spontaneous pneumothorax; the patients with recurrent pneumothorax were comprised of secondary spontaneous pneumothorax patients and those of majority secondary spontaneous pneumothorax patients were observed with bullous emphysema profile. By looking at the pertinent literature, there are publications showing VATS with the recurrence rate ranging from 2% to 14% and post thoracotomy recurrence rate from 0% to 7%. We think that applying pleurectomy, mechanical abrasion and chemical pleurodesis additional to bullae ligation or apical resection in pneumothorax surgery will significantly reduce the recurrence rate.

## 1. Background 

Spontaneous pneumothoraxes constituted 1/1000 hospital admissions. They are particularly one of life threatening health issues in combination with bilateral pneumothorax, tension pneumothorax, respiratory failure or COPD. Primary spontaneous pneumothoraxes within a young population may lead to losses in labor force and economy. Secondary spontaneous pneumothoraxes, more common in the elderly population, could further complicate the underlying disease by adversely affecting the course of their treatment. The recurring nature of pneumothoraxes further emphasizes the importance of the need to take remedial action.

## 2. Objectives 

The cases of spontaneous pneumothorax represent a significant portion of the patients profile within the chest surgery clinics. The risk of recurrent pneumothorax in post thoracoscopy is between 2% and 14%, thus the subject of cure treatment and approach is still controversial. The cases were retrospectively treated due to spontaneous pneumothorax and their reasons, treatment approaches and results were comparatively examined with the literature.

## 3. Patients and Methods

The dates from January 2007 to 2010 79 patients, admitted with the diagnosis of spontaneous pneumothorax to our clinic, were included in the study. The patients’ age, gender, symptoms, held hemithorax, smoking habits, surgical methods applied, results and complications were evaluated. For numerical variables, Mean ± standard deviation were used. For categorical variables % was given. Themes were divided into two groups as PSP and SSP. Mann- Whitney U and Kruskal-Wallis test was used for statistical comparisons between the groups. For statistical perspective P < 0.05 was considered significant. SPSS 20.0 program was utilized for all the analyses.

## 4. Results

**Figure 1:**
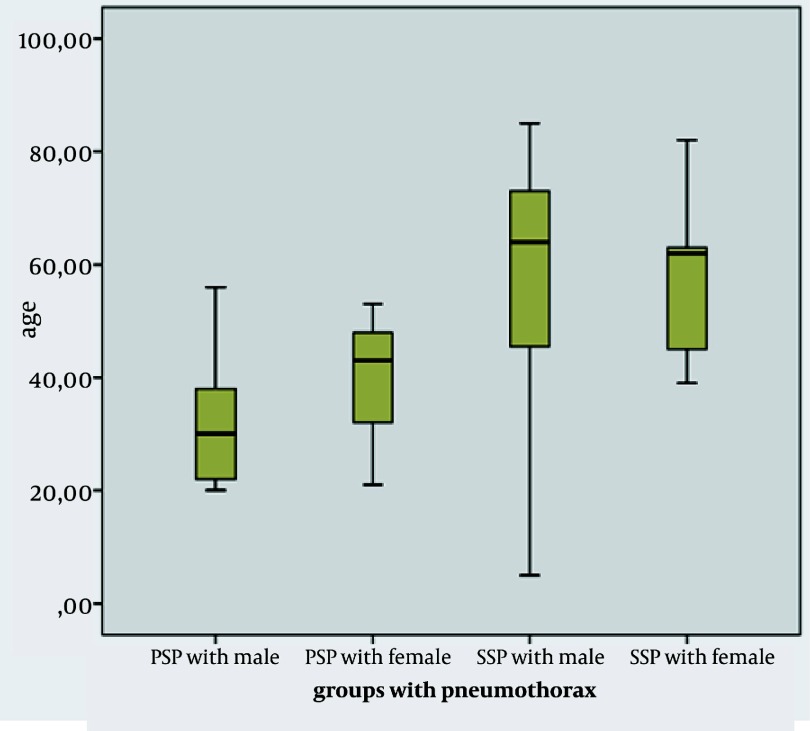
All Patients with Spontaneous Pneumothorax by Age Distribution

Seventy of all the patients (88.6%) were male and 9 of all the patients (11.4%) were female. The mean age was at 45.50 ± 21.07, median; 42 (5-85 ages) (Figure 1). Primary and secondary spontaneous pneumothorax cases were statistically compared with the Mann-Whitney U test. In comparing the distribution of age between the two groups P < 0.05 was considered statistically significant (P = 0.00) (Table 1) .Concentration in primary spontaneous pneumothorax patients was between 20-45 years old (mean; 25.14), for patients with secondary spontaneous pneumothorax were 50-70 years old (mean; 53.77). 38 (48.1%) out of 79 spontaneous pneumothorax patients were the primary spontaneous pneumothorax cases and 41 (51.9%) patients were with the secondary spontaneous pneumothorax cases. 38% of the secondary spontaneous pneumothorax cases were pneumothorax caused by COPD. Table 2 presents the distribution of diseases. COPD was the underlying reason for lung disease in the majority of the secondary spontaneous pneumothorax patients. 24 out of 30 COPD patients were active smokers. 64 of all the cases (81%) were found to be smokers. When each of the two groups was compared regarding smoking, P = 0.017 was found statistically significant. According to this, the secondary spontaneous pneumothorax cases showed a higher rate of smoking habits (Table 1). Pneumothorax was developed in right hemithorax of 43 patients (59.5%) and in left hemithorax of 35 patients (%39.3). Right hemithorax was found in 15 male patients with primary spontaneous pneumothorax, left hemithorax was seen in 18 male patients with primary spontaneous pneumothorax and right hemithorax was detected in 4 female patients with primary spontaneous pneumothorax. 23 male patients with secondary spontaneous pneumothorax developed pneumothorax at their right hemithorax. Although 13 male patients with secondary spontaneous pneumothorax were seen with pneumothorax at their left hemithorax, 5 female patients with secondary spontaneous pneumothorax developed pneumothorax at their right hemithorax (Table 3). Both of the two hemithoraces’ ratio were not found statistically significant in male patients with primary and secondary spontaneous pneumothoraxes (P = 0.108) (not shown). (This comparison using by Kruskal-Wallis test) However, right hemithorax was discovered in 9 female patients with primary and secondary spontaneous pneumothoraxes. This situation was seen as a random result and depended upon the less female patients being in the sample. Table 3 shows this in detail. Patients were admitted with complaints as: 37 (46.8%) with chest pain and dyspnea; 14 (17.7%) with dyspnea; 24 (30.4%) with chest pain; 3 (3.8%) with coughing and 1 (1.3%) with hemopthesis. Patients were checked with a routine physical examination and chest radiography. All the patients were first approached with the tube thoracostomy as their pneumothorax was found above 15%. 39 patients (49.4%) had parsiyel and 40 patients (50.6%) developed total pneumothorax. Of the patients with spontaneous pneumothorax, 55 cases (69.6%) with first attack and 24 cases (30.4%) with second or third pneumothorax attack in post tube thoracotomy were admitted. The patients with the first pneumothorax attack consisted of 25 cases (31.6%) with secondary and 30 cases (38%) with primary pneumothorax. Patients with second or third pneumothorax attack in post tube thoracotomy were 16 cases with secondary and 8 cases with primary pneumothorax. Recurrent pneumothorax in post tube thoracotomy was a higher rate in secondary pneumothorax. An open surgery was applied to the patients with more than a week prolonged air leak and to the patients with second or third pneumothorax attack. Closed underwater drainage with tube thoracotomy to 54 patients, Closed underwater drainage with tube thoracotomy and pleurodesis to 2 patients (2.5%) and Closed underwater drainage with bilaterally tube thoracotomy to 1 patient (1.3%) were performed. 57 of the total patients were approached by tube thoracotomy. 28 F chest drains were used. In 5 patients in post tube thoracotomy due to adhesions massive air leak or bleeding were occurred by the result of parenchymal injury and it was developed more in the secondary spontaneous pneumothorax patients. Tube thoracotomy success rate was 86.1% and recurrence pneumothorax rate in post tube thoracotomy was 13.9%. 22 of the patients were entered through 4 intercostal intervals with axillary thoracotomy. Of the patients treated with thoracotomy, bullae ligation and mechanical abrasion and/or pleurodesis to 6 patients (7.6%), apical resection + apical pleurectomy + tetracycline/talc pleurodesis to 16 patients (20.3%), were applied (Table 4). Bullae ligation was used for single and small bullae detected cases. None of the cases developed recurrent pneumothorax in both open surgical methods within the 1-to-3 year follows up period. The average length of hospital stay was 8.4 ± 5.9, in both groups the average length of stay in hospital was found statistically significant. (P = 0.00) The average length of stay in hospital for secondary spontaneous pneumothorax was longer. Post thoracotomy complication was not encountered. (Table1). Mortality was one patient (1.3%), who was admitted for developing secondary pneumothorax with hemoptysis in stage 4 lung cancer cavitary lesion. 4 of the patients (3.2%) had a family history of spontaneous pneumothorax.

**Table 1:** Distrubition of Groups with Pneumuthorax According to the Demographic and Post-operative Data

	Patients with PSP^a^	Patients with SSP ^a^	Total (n = 79), No. (%)	P value^b^
**Localization of pneumothorax**				0.08
Right	15	28	43 (59.5)	
Left	22	13	35 (39.3)	
Mean rank	43.92	36.37		
Bilateral	1	0	1 (1.2)	
**Smoking habit**				0.017
Smoking	29	35	64 (81)	
Mean rank	35.66	44.02		
Non smoking	9	6	15	
**The average length of stay post-operative**				0.00
Day length	5.63	10.9	8.4	
Mean rank	29.22	49.99		
**Age, y, Mean**	25.14	53.77	45.50	0.000

^a^Abbreviation: PSP, primary spontaneous pneumothorax; SSP, secondary spontaneous pneumothorax

^b^P value, Mann-Whitney U Asymp. Sig. (2-tailed) analysis was performed

**Table 2:** Secondary Spontaneous Pneumothorax Patients’ Distribution According To Gender and Diseases

Diseases	Patients’ Gender		Total, No. (%)
	Female	Male	
**COPD**	1	29	30 (38)
**Interstitial lung disease**	1	1	2 (2.5)
**Infection**	1	4	5 (6.3)
**Lung Cancer**	1	3	4 (5.1)

**Table 3:** Right and Left Hemithoraxes Held In Spontaneous Pneumothorax Patients

Spontaneous Pneumothorax	Patients, No.	Patients’ mean age, y	patients with Right Pneumothorax	patients with Left Pneumothorax	patients with Bilateral Pneumothorax
**Primary SP **^a^	38				
Female	4	30.25	4	0	
Male	34	30.78	15	18	1
**Secondary SP**	41				
Female	5	58.20	5	0	
**Male**	36	60.70	23	13	

^a^abbreviation: SP, spontaneous pneumothorax

**Table 4:** The Distribution of Surgical Approaches in Cases of Secondary and Primary

Surgical Procedures	Patients with secondary spontaneous pneumothorax, No.	Patients with primary spontaneous pneumothorax, No.	Total, No.
**Tube thoracotomy**	22	32	54
**Tube thoracotomy and pleurodesis**	2	0	2
**Thoracotomy-bullae ligation and mechanical abrasion**	4	2	6
**Thoracotomy-apical resection-apical pleurectomy and tetracycline/talc pleurodesis**	13	3	16

## 5. Discussion

The accumulation of air without trauma in pleural space is defined as spontaneous pneumothorax. If pneumothorax is developed due to underlying a lung disease, it is secondary and developing pneumothorax without a lung disease is called primary spontaneous pneumothorax (1-3). Bullae and blebs are important factors in the formation of spontaneous pneumothorax (4). Blebs are the accumulation of small sub pleural air that is caused by alveolus rupture creating air gathering inside visceral pleura. Blebler are separated from the normal parenchymal tissue in a clear lining and they are connected with a narrow neck to the parenchymal tissue. In general, they are either in the apex of the upper lobes or in superior segment of the lower lobes. Blebler, as periasiner or paraseptal emphysema, are thought to be a form of interstitial emphysema (4). Bullae are the wide spaces filled with air. Alveolar are formed by the result of wall destruction. They are linked with any form of emphysema (5). The pleural cavity rupture has been reported due to bullae and blebs increasing over a certain alveolus pressure. In 1980 Ohata and his/her colleagues reported in their study that pleural mesothelial cell structures were differentiated in three types of emphysema and that without an effect of bullae rupture there was air leakage in a certain pressure from bullae wall to the intrapleural cavity (6). Secondary spontaneous pneumothorax is most commonly developed in COPD (68%), tumors (16.9%), sarcoidosis (4.7%) and pulmonary tuberculosis (1.6%). (1, 5, 7, 8). In our series, COPD- related pneumothoraxes were 38%. Çok G et al. (8) in their series of 53 spontaneous pneumothorax patients were reported with COPD (42%) as the most frequently underlying lung disease. COPD related deaths are in 3 rank in the world and the incidence of COPD related pneumothoraces is at 26/100000 (9, 10). COPD related 1 in 4 patients with under 1 litre FEV1 FEV1 / FVC rate under 57% studies published a development of pneumothorax (1, 10).The incidences of primary and secondary spontaneous pneumothorax were reported as 6.3/100000 in men and 2/100000 in women per annum (8). Spontaneous pneumothorax rate of hospitalization is 1/1000 (11). Risk factors were presented as tall and asthenic structure, smoking habits, genetic connective tissue diseases of familial predisposition like marfan syndrome or ehlers danlos syndrome, changes in atmospheric pressure, bronchial anomalies in the etiopathogenesis of primary spontaneous pneumothorax. A family history of pneumothorax was present at 4% in our cases (12). The difference in Mean age for each of the two groups was found statistically significant. mean age related primary spontaneous pneumothorax 20-45 years old cases was 31.5, mean age secondary spontaneous pneumothorax related 50-70 years old cases, it was found that the concentration was on the age of 58.4. In both groups, the male patients were seen 7-8 times more than female patients. İt is compatible with literature data (4, 11). Smoking habit rate was higher with secondary spontaneous pneumothorax cases and a statistically significant difference was found in comparing both of the two groups. Pneumothorax surgical treatments are consisted of closed tube thoracotomy with underwater drainage or thoracentesis and high nasal oxygen therapy, VATS or thoracotomy with bullae, blebs resection, apical resection and pleural procedures. In our study, recurrence rate in post tube thoracotomy was 13.9%, and success rate was at 86.1%. Complication rate in post tube thoracotomy was found at 6.3%. In Hong Kong, the multi-centered pneumothorax cases’ tube thoracotomy success rate was published as being 77%. A surgery was applied to 8% of all the cases (13). Pneumothoraxes treated within thoracic surgery disease group come in second rank after the most common form of lung cancer. Although the recurrence rate in post pneumothoraxes surgery is high, but a cure treatment has not been found yet. Formation of the new bullae together with age could not be prevented in occurring (14). In order to prevent and reduce the recurrence rate, one of these procedures is recommended namely mechanical abrasion, parietal pleuretomy and chemical pleurodesis. Parietal pleuretomy is superior to the other two procedures. Recurrence rate is reported as 0.48% in post parietal pleuretomy, 7% in pleurodesis with talc and 8-25% in tetracycline pleurodesis (3,7, 15). In our researched series, intraoperative mechanical abrasion to 6 patients, and pleuretomy and pleurodesis to 16 patients were applied. Recurrence in post VATS is 4-20% higher than recurrence in post thoracotomy (16). Recurrence rate is high in elderly patients and persistent pneumothoraces are being increased. In 1990s, Video assisted thoracic surgery due to its minimal invasiveness was a heavily preferred approach in pneumothorax surgery. It is reported that recurrence rates were higher than expected. There are publications indicating that 90% of the patients treated with thorascoscopic blebektomi, but not the ones treated with pleurodesis, within the first two years, recurrence rate, especially in young bilateral spontaneous pneumothorax patients’ group, has much developed new bül formation in postoperative stapler line, and recurrent pneumothorax rate is higher with traditional surgery method (17). According to the data from the literature, in order to reduce the recurrent rate in primary spontaneous pneumothorax and video assisted thoracic surgery, apical resections as well as apical pleurectomy procedures are recommended (18). In older patients with bad general health state as well as with persistent pneumothorax, air leakage is controlled by applying fibrin glue bullae as torakografik in an invasive radiological initiative; towards to reduce recurrence rate in postoperative and persistent SSP related cases Total Pleural Covering method with sticking an oxidize cellulosic mesh fibrin glue to support the staple line, just in case needed later, adhesionness to the chest wall in thoracic surgeries are reported less than other agents (14). By adding apical resection and apical pleurectomy, recurrence rate being reduced to 0.48% was published in the literature data (19). Weeden, Smith and their colleagues (20) informed that complication rate in post apical pleurectomy was 9.5%, the recurrence rate in post pleurectomy was 0.43%. In our study, 22 out of 79 patients received the classic surgical method and 16 of these patients were given apical pleurectomy with 500mg tetracycline and chemical pluerodesis. Postoperative hematoma or residual apical pneumothorax was not followed. As Kuzucu and his/her colleagues (21) expressed in their publications, we think that surgical treatment method will be appropriate for the patients of 2recurrence pneumothorax attack. According to the literature data about spontaneous pneumothoraxes, surgical rates show differences. Our working group’s surgical rate is 26%. We think that bullae ligation in pneumothorax surgery or apical resection as well as partial pleural decortication and pleurodesis application in good health condition patients will reduce the recurrence rate. There are publications reporting that total pleurectomy and pleurodesis are the gold standart (7, 22). The limitation of our study was that a small sample of patients was treated with bullae ligation and others including pleural abrasion, apical resection, pleurectomy + pleurodesis as operative methods.
